# Around the Hospitals

**Published:** 1894-03-03

**Authors:** 


### Around the Hospitals.

Notice to the Managers of Institutions.—Special space is now reserved for the insertion of notices of hospital meetings and festivals. The Editor requests that all notices may reach The Hospital Office, 428, Strand, at least one week before the date of each meeting.

**The Edinburgh Royal Infirmary.**—At this hospital the average number of in-patients has risen by 38 during the past year, the pressure in the female wards having been especially great. In the out-patients' department a large increase is also visible, amounting to as many as 2,000 persons. Satisfactory as is the additional income also received, it is to be hoped that efforts will not flag to secure a steadily increasing income, as in the great work of the institution, and having regard to its well-known efficiency, its needs are not likely to diminish. A novel feature, we believe, exists at the Edinburgh Infirmary of selling hospital tickets to students, and from this source an income of £455 was derived during last year. During the same period the provision required by the Triple Qualification Board for the education of female students has been provided at the infirmary, and a large number of beds in female wards are now allotted to them.

**The Royal Free Hospital.**—The past year has been a very favourable one at the Royal Free Hospital, yet, unfortunately, the large income of 1893 cannot be regarded as a reliable basis for future expectancy. In 1891 the total receipts amounted to £7,751, whilst in 1893 they reached £14,431. The total ordinary receipts in 1893 show a diminution, however, and the expenditure more than doubled these receipts. Therefore, whilst congratulating the hospital on the very large legacies they received, amounting to £9,167, we would urge the public not to overlook the fact that the institution requires a far larger amount of support from annual subscriptions than formerly, seeing that in spite of the temporary closing of one of the wings, the ordinary income amounted to half the sum expended. The rebuilding of the front of the hospital has been commenced, and of the £12,000 to complete the work appealed for by Lord Dufferin but £7,000 now requires to be raised. Already the large boilers required for the laundry are fixed in the basement of the building, and it is hoped that the new buildings will be completed by the autumn of the present year. The number of in-patients received in 1893 amounted to 1,554, and the out-patients to 24,736. The work of the Samaritan Society will be much increased, owing to Mr. Spicer's legacy amounting to about £10,500, the proceeds of which, when invested, will be devoted to this branch of the hospital's work. Two fires occurred in the hospital during the year, but they were quickly extinguished through the prompt action of the steward, nurses, and officers, who are trained to render assistance in case of fire.

**The Bradford Infirmary.**—The infirmary has had an unusually hard time during the past year. The in-patients increased 20 per cent., and the closing of the fever hospital to all but small-pox cases caused the introduction of scarlatina patients, who had to be transferred to their homes, but not before the disease was spread in the infirmary, the infection being eliminated at much trouble and expense. It is difficult to estimate where the consequences of authorities neglecting to provide proper fever accommodation, as at Bradford, end. Financially, the year has been successful. A good number of legacies have fallen in, and some generous donations have been made. Annual subscriptions show a slight diminution, which diminution the Mayor's appeal to those who have benefited through the institution will, it is to be hoped, turn into a surplus for the next annual record. The work of the infirmary is largely carried on by the Woodlands Convalescent Home, which received 854 patients during the year. In the infirmary itself many improvements are projected which will add to the efficiency and comfort of the institution. The number of in-patients received amounted to 1,937, and out-patients numbered 2,241. The total income was £10,185, and the expenditure £9,041.

